# Pulmonary Valve Regurgitation: Neither Interventional Nor Surgery Fits All

**DOI:** 10.3389/fped.2018.00169

**Published:** 2018-06-07

**Authors:** Antonio F. Corno

**Affiliations:** ^1^East Midlands Congenital Heart Centre, University Hospitals of Leicester, Leicester, United Kingdom; ^2^Cardiovascular Research Center, University of Leicester, Leicester, United Kingdom

**Keywords:** congenital heart defects, pulmonary valve regurgitation, pulmonary valve implantation, percutaneous invitation, pulmonary artery

## Abstract

**Introduction:** PV implantation is indicated for severe PV regurgitation after surgery for congenital heart defects, but debates accompany the following issues: timing of PV implantation; choice of the approach, percutaneous interventional vs. surgical PV implantation, and choice of the most suitable valve.

**Timing of pulmonary valve implantation:** The presence of symptoms is class I evidence indication for PV implantation. In asymptomatic patients indication is agreed for any of the following criteria: PV regurgitation > 20%, indexed end-diastolic right ventricular volume > 120–150 ml/m^2^ BSA, and indexed end-systolic right ventricular volume > 80–90 ml/m^2^ BSA.

**Choice of the approach: percutaneous interventional vs. surgical:** The choice of the approach depends upon the morphology and the size of the right ventricular outflow tract, the morphology and the size of the pulmonary arteries, the presence of residual intra-cardiac defects and the presence of extremely dilated right ventricle.

**Choice of the most suitable valve for surgical implantation:** Biological valves are first choice in most of the reported studies. A relatively large size of the biological prosthesis presents the advantage of avoiding a right ventricular outflow tract obstruction, and also of allowing for future percutaneous valve-in-valve implantation. Alternatively, biological valved conduits can be implanted between the right ventricle and pulmonary artery, particularly when a reconstruction of the main pulmonary artery and/or its branches is required.

**Hybrid options: combination of interventional and surgical:** Many progresses extended the implantation of a PV with combined hybrid interventional and surgical approaches. Major efforts have been made to overcome the current limits of percutaneous PV implantation, namely the excessive size of a dilated right ventricular outflow tract and the absence of a cylindrical geometry of the right ventricular outflow tract as a suitable landing for a percutaneous PV implantation.

**Conclusion:** Despite tremendous progress obtained with modern technologies, and the endless fantasy of researchers trying to explore new forms of treatment, it is too early to say that either the interventional or the surgical approach to implant a PV can fit all patients with good long-term results.

## Introduction

The pulmonary valve (PV) regurgitation following the surgical repair of congenital heart defects is generally tolerated quite well in the immediate post-operative period and for the first few years (1). However, on longer-term clinical observations the presence of severe PV regurgitation can be associated with exercise intolerance, heart failure, ventricular arrhythmias with syncope episodes and sudden death (2–10). These symptoms are related to right ventricular volume and/or pressure overload, inducing dilatation and dysfunction, and ultimately right ventricular failure (5, 8, 9, 11); sometimes the clinical conditions are further deteriorated by the subsequent impairment of the left ventricular function (8).

PV implantation reduces the right ventricular end diastolic volume, preventing pathological right ventricular remodeling, and restores the right ventricular function, preventing progressive dilatation toward right ventricular failure (7, 9, 11–16).

With regard to the need for a PV implantation in the presence of severe PV regurgitation, extensive debates accompany the following issues:

- timing of PV implantation, particularly in asymptomatic patients (5–7, 9, 13– 24)
- choice of the approach, percutaneous interventional (5, 25–38) vs. surgical (7, 9, 11–18, 39–44) PV implantation
- choice of the most suitable valve (2, 39–43, 45–53).

## Timing of pulmonary valve implantation

In the presence of PV regurgitation, the presence of clinical symptoms clearly corresponds to class I evidence of indication for PV implantation in both the European (19) and North-American (20) guidelines (5, 7, 9, 13–18, 21–24).

In asymptomatic patients the criteria for indications for PV implantation, and particularly the choice of the best timing, are less clearly defined (54). General agreement exists on the indication in asymptomatic patients in the presence of any of the following criteria, as judged by echocardiography and/or magnetic resonance imaging (13, 17, 18, 21, 23, 24):

- PV regurgitation > 20%
- indexed end-diastolic right ventricular volume >120–150 ml/m^2^ BSA
- indexed end-systolic right ventricular volume > 80–90 ml/m^2^ BSA

## Choice of the approach: percutaneous interventional vs. surgical

The choice between the approach with percutaneous interventional (5, 25–38) vs. surgical (8, 9, 11–18, 39–43) PV implantation is generally the result of a decision making process taking into consideration:

- morphology and size of the right ventricular outflow tract, in particular in the presence of a previously implanted trans-annular patch
- morphology and size of the pulmonary arteries in the case a required enlargement or reconstruction
- presence of residual intra-cardiac defects
- presence of extremely dilated and dysfunctional right ventricle (52, 55–57)

If reconstruction of the pulmonary artery branches, or closure of residual intra-cardiac defects, or remodeling of a hugely dilated right ventricle, are required in association with the PV implantation, and these cannot be accomplished by a procedure of interventional cardiology, then the indication is given for surgical implantation of a PV or a right-ventricle to pulmonary artery valved conduit (7, 9, 11–18, 39–44).

## Choice of the most suitable valve for surgical implantation

Regarding the choice of the most suitable valve to implant in PV position, the first choice is not for a mechanical prosthesis, despite the proven longevity, because of the risks of thromboembolic phenomena and the requirement for life-long anticoagulation (41, 48), with tissue valves considered as the first choice for PV implantation (2, 39–49). Despite the various models of biological valves available for implantation in PV position, the optimal valve choice remains unknown, partly because long-term out-comes are deficient owing to structural valve deterioration necessitating further treatment (48).

The Trifecta® (Abbott, Abbott Park, Illinois, USA) valve, tri-leaflet stented pericardial valve designed for implantation in aortic position, with a supra-annular sewing cuff, pericardial-covered posts, pericardial leaflets taking origin from the exterior of the valve construct, increasing the effective orifice area for any external diameter, provided good results in PV position (51, 52).

These characteristics should provide a reduced rate of structural valve deterioration over time, particularly because previous studies showed that PV implantation performed in young patients, with relatively smaller size biological valves, correlated with a higher risk of valve failure and need for early valve re-replacement (45, 46).

Regarding the size of the valve to implant in PV position, our choice is to implant a relatively large size of the biological prosthesis, adequate for the effective orifice area of the native PV indexed for the patient BSA (52). This choice presents the advantage not only of avoiding a right ventricular outflow tract obstruction, but also of allowing for future percutaneous valve-in-valve implantation in the case of degeneration of the currently implanted valve (52). Of course we very carefully avoided oversizing the valve, because of the well-known reduced duration of oversized biological valves.

Alternatively, biological valved conduits, such as homografts, bovine jugular veins, or Hancock® conduits (Medtronic, Minneapolis, Minnesota, USA), can be used as PV, with the implantation accomplished between the right ventricle and pulmonary artery. As this surgical approach is more extensive than the simple PV implantation, the choice of right ventricle to pulmonary artery biological conduit for PV regurgitation is particularly utilized when the reconstruction of the main pulmonary artery and/or its branches is required (50, 53, 58).

## Hybrid options: combination of interventional and surgery

Since the first clinical report of interventional implantation of a pulmonary valve in a right ventricle to pulmonary artery conduit with valve dysfunction (59) and our report of the first of-bypass surgical implantation of a pulmonary valve of large size, through a purse string on the anterior surface of the right ventricle, in an experimental model (60), many progresses have been made to extend the implantation of a pulmonary valve with combined hybrid interventional and surgical approaches, with generally good results (61–68).

The major efforts have been made to extend the PV implantation in the presence of dilated right ventricular outflow tract, previously the major limit to the use of interventional technique (63–67). These included pre-stenting in preparation for the later implant of the biological valve (63), simultaneous pre-stenting and implantation (64), valve implantation after a PTFE graft positioning in the dilated right ventricular outflow tract with surgical approach on cardiopulmonary bypass, using the PTFE graft as landing site for the valve (65), and surgical implantation of a percutaneous biological valve after placing a 4 mm PTFE graft wrapped around the valve (67).

All the above reported techniques, very recently reported in the literature, clearly express the attempt of overcoming the current limits of percutaneous PV implantation, namely the excessive size of a dilated right ventricular outflow tract and the absence of a cylindrical geometry of the right ventricular outflow tract as a suitable landing for a percutaneous PV implantation.

## Conclusion

Despite the tremendous progress obtained with the availability of modern technologies, and the endless fantasy of researchers trying to explore new forms of treatment, at the moment it is too early to say that either the interventional or the surgical approach to implant a PV can fit all patients with good long-term results.

## Author contributions

AC provided the conception and design of the article, drafted the work and revised for the content, and agreed to be accountable for all aspects of the work.

### Conflict of interest statement

The author declares that the research was conducted in the absence of any commercial or financial relationships that could be construed as a potential conflict of interest.
